# Spatially explicit power analysis reveals challenges for a long‐term threatened species monitoring program in Australia

**DOI:** 10.1002/eap.70271

**Published:** 2026-06-05

**Authors:** Vishnu Menon, Darren Southwell, Alan Robley, Matthew W. Rees, David P. Wilkinson, Katherine Giljohann, Jack Pascoe, Brendan Wintle, Bronwyn A. Hradsky

**Affiliations:** ^1^ School of Agriculture, Food and Ecosystem Sciences University of Melbourne Melbourne Victoria Australia; ^2^ School of Environmental and Life Sciences University of Newcastle Newcastle New South Wales Australia; ^3^ Department of Energy, Environment and Climate Action Arthur Rylah Institute for Environmental Research Heidelberg Victoria Australia; ^4^ CSIRO Land and Water, Commonwealth Scientific and Industrial Research Organisation Brisbane Queensland Australia; ^5^ CSIRO Land and Water, Commonwealth Scientific and Industrial Research Organisation Melbourne Victoria Australia

**Keywords:** Australia, baiting, distribution modeling, feral cat, invasive predator, long‐term monitoring, red fox, statistical power, threatened species

## Abstract

Long‐term monitoring programs are crucial to assess trends in biodiversity and so make informed decisions for conservation and resource management. However, disregarding the statistical power of a monitoring program can lead to incorrect conclusions about species population trends, potentially resulting in ineffective management and misdirected resource allocation. In Australia, predation by introduced red foxes (*Vulpes vulpes*) and feral cats (*Felis catus*) remains a major cause of native faunal decline and extinction. Australia spends more than $16 million yearly in controlling foxes for biodiversity conservation, primarily through landscape‐scale poison baiting. Using a long‐term fox baiting and threatened species monitoring program in southeastern Australia, we collated data from 2132 camera‐trap deployments to: (1) explore drivers of the distribution of threatened native mammals and introduced predators, (2) conduct a spatially explicit power analysis to assess the program's ability to detect trends in native and introduced species occupancy for the next 10 years, and (3) provide recommendations for improving monitoring efforts through alternative scenarios. We found that threatened native mammals were more likely to occupy areas with high densities of fox baits, whereas foxes were less likely to occupy these areas; however, these areas were quite localized within baited regions. The power of the existing monitoring design was sensitive to the magnitude of change in occupancy, but robust to approximately 15% changes in the number of survey sites. The monitoring program showed adequate power (>0.8) to detect its original aims: increases in threatened native mammal occupancy and decreases in fox occupancy in baited areas. Hence, the lack of a strong signal of increasing native mammal occupancy in the last 8 years likely indicates that the system has reached a stable state under current management, rather than poor statistical power. This may potentially be the case in many long‐term predator management programs. If removing some sites from an existing monitoring design does not considerably vary power, managers could consider diverting these resources to, for example, improving understanding of species–habitat relationships or intensifying predator management efforts.

## INTRODUCTION

In recent decades, the decline and extinction of numerous faunal species globally (Ceballos et al., 2017; Woinarski et al., 2015) has underscored the importance of long‐term monitoring programs (Lindenmayer et al., 2022; Wintle et al., 2019). Long‐term monitoring programs are also important for observing and managing changes in ecosystems, as ecological processes often extend beyond the typical time frames of conventional environmental measurements (Giron‐Nava et al., 2017; Owens, 2013). Hence, there has been growing recognition of the value of long‐term monitoring (Lindenmayer et al., 2022).

Designing large‐scale monitoring programs requires making decisions about when and where to monitor, which species are of interest, the types of data to be collected (e.g., counts, abundance), the types of monitoring method (e.g., camera monitoring, spotlighting) and frequency of monitoring (Einoder et al., 2018; Field et al., 2005; Southwell et al., 2019). These decisions can be constrained by financial challenges (Nichols & Williams, 2006; Timofeyev, 2016) or lack of clear objectives (Lindenmayer & Likens, 2009); resulting in poor experimental designs or monitoring frameworks that are too generic for suite of taxa or ecosystems being considered (Lindenmayer & Likens, 2018).

Power analyses can help guide the design of monitoring programs and evaluate trade‐offs in sampling effort to achieve a high probability of detecting biologically significant effects (Field et al., 2007; Steidl et al., 1997). Statistical power (hereafter “power”) refers to the probability that a given monitoring program can detect a statistical change, if there is an effect of a given size (Guillera‐Arroita & Lahoz‐Monfort, 2012). Disregarding the power of a monitoring program may result in incorrect conclusions about the trends in species populations, potentially causing ineffective management and misdirected resource allocation (Einoder et al., 2018). In addition, the power of a monitoring program may differ over time in response to changes in species prevalence and detectability, or changes in survey effort (Guillera‐Arroita & Lahoz‐Monfort, 2012). Hence, evaluating the statistical power of existing monitoring programs is important (Southwell et al., 2019). Power analyses of existing programs can also help land managers make appropriate decisions on whether to reallocate existing resources to less surveyed areas to improve power, or reduce survey efforts if the power of the monitoring design is strong and resources are limited.

Camera traps are increasingly recognized as a standard, cost‐effective method for long‐term monitoring, with the capacity to detect species that are elusive and have low detection rates (Grotta‐Neto et al., 2020; Linden et al., 2017) and concurrently monitor an assemblage of species (Kays et al., 2020). Camera trap data can be used to monitor how species occupancy, diversity, and/or relative abundance vary across spatial and temporal scales (O'Connell et al., 2011). However, the rapid growth of camera trap surveys for monitoring has led to subsequent variations in study designs, with monitoring programs often overlooking appropriate experimental setup or optimization of field methods (Burton et al., 2015; Kays et al., 2020; Nawaz et al., 2021).

The power of a monitoring program to detect a change in species occupancy over time varies with the species’ occupancy (proportion of sites where a species is found) and detectability (probability of detecting a species at a site given it is present), as well as the sample size, effect size, monitoring methods, and survey effort (Guillera‐Arroita & Lahoz‐Monfort, 2012; Johnson et al., 2015). Recent frameworks for power analyses for occupancy monitoring can help incorporate spatially explicit strategies to optimize monitoring design strategies (Ellis et al., 2014; Smart et al., 2022). Spatially explicit power analyses allow for the spatial arrangement of sites relative to the distribution of target species to be considered by land managers when designing monitoring programs for multiple species over a landscape scale (Southwell et al., 2019).

In Australia, predation by introduced red foxes (*Vulpes*; hereafter “fox”) and feral cats (*Felis catus*; hereafter “feral cat”) has resulted in decline and extinction of numerous endemic terrestrial fauna since European colonization (Woinarski et al., 2015). Hence, landscape‐scale introduced predator control is widely applied as a conservation management strategy (Comer et al., 2020; Saunders et al., 2010). In some cases, long‐term monitoring programs have been established alongside these control programs, but the statistical power for these long‐term monitoring programs in detecting species trends is often not tested during establishment or as more data are collected over time.

Here, we use a long‐term threatened species monitoring and predator management program conducted over 18 years in southeast Australia as a case study to: (1) explore the drivers affecting the distribution of threatened native mammals and introduced predators, (2) conduct a spatially explicit power analysis to evaluate the statistical power of the monitoring program to detect changes in species occupancy over time, and (3) develop recommendations for improving monitoring effort by assessing alternate realistic monitoring scenarios. A similar framework could readily be applied to other long‐term occupancy monitoring programs associated with a control‐treatment management program.

## METHODS

### Study area and data collection

The Glenelg Ark program in southeast Australia was set up in 2005 to facilitate the recovery of native mammals from predation by foxes. It includes a robust management and monitoring design with replicated control‐treatment pairs across three treatment areas (fox baited; size 4703–9750 ha each) and three nontreatment areas (unbaited; size 4659–8520 ha each) that are spatially independent (Robley et al., 2014). Fox baiting treatments are consistent across years, with baits containing 3 mg of sodium monofluoroacetate (1080) poison buried at 1‐km intervals along roads and tracks. Bait densities vary from 0 to 1.26 baits per km^2^, due to differences in road networks among treatment areas. Annual monitoring is carried out at 40 sites within each treatment and nontreatment area. Sites were selected by stratifying ecological vegetation classes based on the habitat requirements of the target species (Robley et al., 2011). Camera‐trap monitoring at these sites commenced in 2013. Camera‐trap monitoring was replacing hair tube surveys used for monitoring native mammals from 2005 to 2013.

Continuous landscape‐scale fox baiting is carried out to primarily facilitate the recovery of two threatened ground‐dwelling native mammals that are of key conservation interest for Glenelg Ark: southern brown bandicoots (*Isoodon obesulus*; hereafter “SBB”) and long‐nosed potoroos (*Potorous tridactylus*; hereafter “LNP”). These two medium‐sized native mammals (450–5000 g) occur in patchy distributions and low abundance in parts of southeast Australia (Menkhorst & Bennett, 1995; Robley et al., 2011). Both species are frequently found in diets of foxes (Hradsky, Mildwaters, et al., 2017; Triggs et al., 1984) and have been observed to increase in fox‐controlled areas (Dexter & Murray, 2009; Robley et al., 2014).

After 8 years of management, Robley et al. (2014) found a substantially reduced bait uptake by foxes in treatment areas, and increasing native prey occupancy in baited areas compared to nontreatment areas. However, the response to ongoing fox baiting by SBB and LNP in baited areas within the Glenelg Ark program has not considerably improved in the following 10 years, and there is a potential concern of feral cat increase in fox‐baited areas due to a potential “mesopredator release” (Rees et al., 2023; Robley et al., 2023). Mean naive occupancy plots in treatment and nontreatment areas from camera trap data plotted in Appendix S1: Figure S3 show no consistent increase in threatened native mammal occupancy (<0.25). Land managers are concerned whether these equivocal results truly reflect no change (perhaps due to SBBs and LNPs reaching their natural carrying capacity or being limited by feral cat predation), or whether it would be beneficial to reallocate or increase survey effort to improve the power of the monitoring design.

**FIGURE 1:**
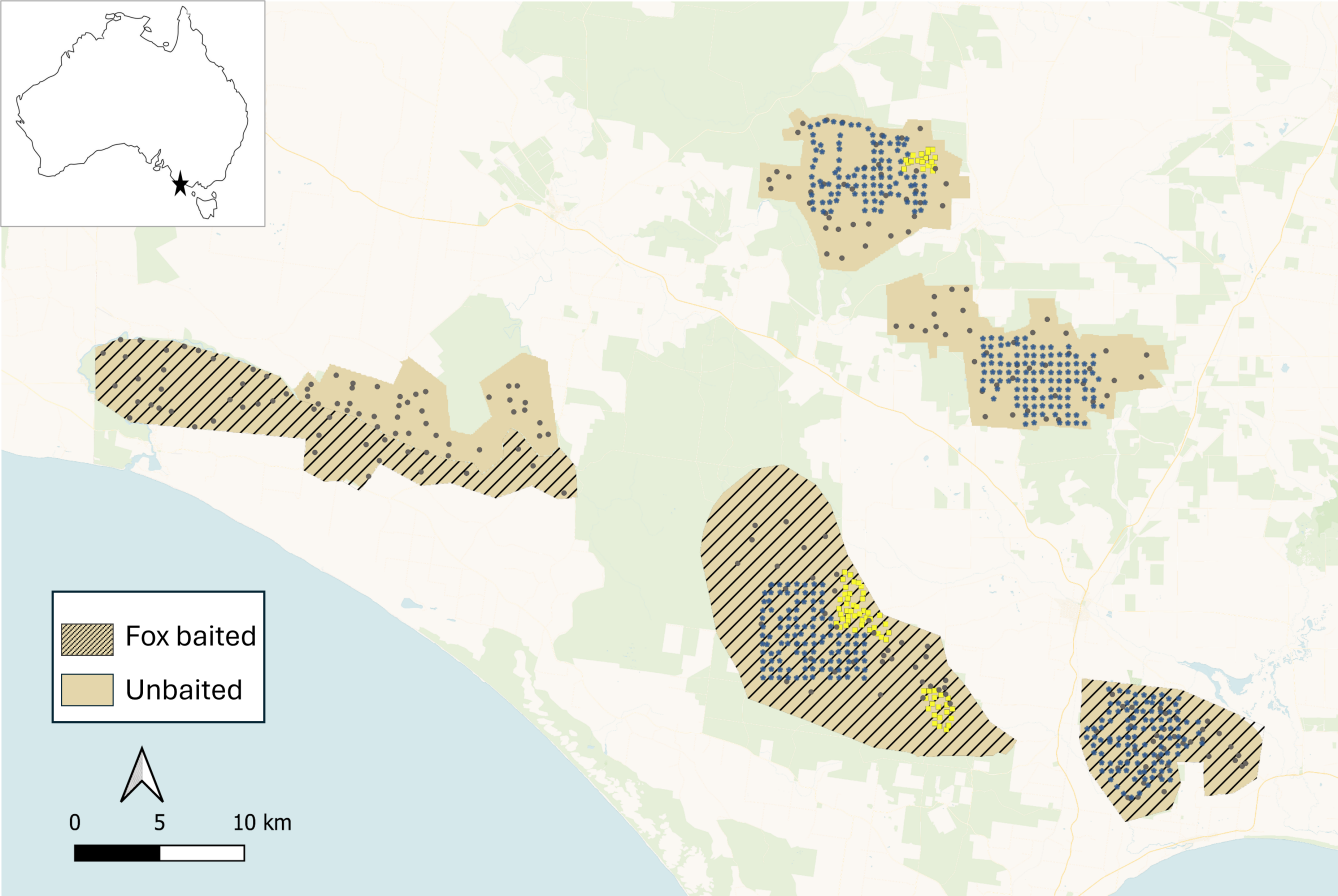
Camera monitoring sites for yearly monitoring by Glenelg Ark (black dots), sites from Rees et al. (2023) (blue stars) and from Menon et al. (2025) (yellow squares) in the fox‐baited (shaded) and unbaited (unshaded) for Glenelg Ark. This totaled 2132 distinct camera trap deployments between 2013 and 2020. Native vegetation is indicated by pale green shading, and major roads are shown as brown lines.

Spatially explicit power analyses require occupancy and detectability raster maps for each species for the simulation framework developed by Southwell et al. (2019). To generate these maps, we collated data from multiple camera‐trap studies in the Glenelg region: 240 sites that are monitored yearly as part of Glenelg Ark (total of 1624 deployments, data sourced from 2013 to 2019), 425 sites that were surveyed once (Rees et al., 2024), and 83 sites surveyed once (Menon et al., 2025). This totaled 2132 camera‐trap deployments between 2013 and 2020 (Figure 1). Rees et al. (2024) assembled a similar dataset; however, they did not predict landscape‐wide species occupancy and detectability across Glenelg Ark and used generalized additive models that did not account for imperfect detection. Reconyx motion‐sensored cameras (RapidFire HC600 or Hyperfire HF2X) were used for all studies. All studies used one camera per site. The camera was fastened on a tree at a height of approximately 30 cm above the ground, facing towards a lure setup approximately 1.5–2.5 m away. Robley et al. (2023) used a lure of truffle oil, peanut butter, rolled oats, and golden syrup in a small, ventilated canister secured to the ground. A similar lure was used for our data (Menon et al., 2025), but the lure was contained within two tea strainers fastened to a stake at a height of 40–60 cm above the ground. Rees et al. (2024) used a lure of a cloth soaked in tuna oil within a small, ventilated canister; white feathers were attached to the outside of the canister, which was fastened near the top of a 1‐m wooden stake. Camera traps were active for an average of 46 days (range 28–93 days).

We collated data on two threatened native mammals (SBB and LNP) and two introduced predators that are known to prey on ground‐dwelling native mammals in the region (fox and feral cat). While fox baiting is primarily targeted at controlling foxes in Glenelg Ark, we included feral cats as recent studies have shown that feral cat density is higher in some fox‐baited areas (Marlow et al., 2015; Rees et al., 2023). We generated weekly detection histories for each species, with 0 representing a weekly non‐detection and 1 representing a weekly detection. Weeks were defined as 7 × 24‐h periods from midnight after deployment. Cameras needed to be active for at least 5 days within the week to be included in the weekly detection history matrix. The number of camera trapping weeks per survey ranged between 4 and 13 weeks.

### Occupancy‐detection models and raster maps

We extracted data on predictors that could potentially impact occupancy and/or detectability of introduced predators and threatened native mammals in our study region (Appendix S1: Table S1 and Figure S1). These included distance to non‐native vegetation, distance to roads, distance to water, elevation, normalized difference vegetation index (NDVI), topographic wetness index (hereafter “wetness”), bait density, time since fire (TSF), ecological vegetation division, and lure type (peanut butter or tuna oil). We centered and scaled all numeric covariates to have a mean of 0 and SD of 1 using an R function “scale” before extracting predictor values for respective sites. We calculated Pearson's correlation test to test for any strong correlation between the covariates (Appendix S1: Figure S2). All correlations were considered weak (<0.4) and so all covariates were retained (Akoglu, 2018).

Occupancy and detectability were modeled for each species using a hierarchical occupancy‐detection model (MacKenzie et al., 2002) in a Bayesian framework using “ubms” package in R (Kellner et al., 2021). We fitted a “stacked” single season occupancy‐detection model to each species (Kellner et al., 2021), with yearly repeat surveys (2013–2019) considered as new sites and temporal replication addressed by adding “site” as a random effect. We ran a “full” model with bait density, TSF, and lure type affecting detectability, and all predictors except lure type affecting occupancy:

1. Detection ~ Bait density + Time since fire + Lure type.
2. Occupancy ~ Distance to non‐native vegetation + Distance to roads + Distance to water + Elevation + NDVI + Wetness + Bait density + Time since fire + Ecological Vegetation Division + Lure type + (1|site).

Additionally, we fitted a model with only survey year and treatment (categorical baited and unbaited) affecting occupancy (and site as random effect) and constant detectability to test whether they matched the observed naive occupancy trends from Glenelg Ark monitoring (Appendix S1: Figure S3). Models were run with four MCMC chains and 10,000 iterations per chain. Occupancy and detectability values ranged from 0 to 1. We made inferences on the full model and considered the effect of a covariate to be supported if 95% credible intervals around the coefficient did not overlap 0. We also evaluated whether the effect was positive (>0) or negative (<0).

We assessed model goodness of fit using the Mackenzie–Bailey χ^2^ test in the “ubms” package, with mean posterior predictive *p*‐values near 0.5 considered a good fit (values range from 0 to 1; Kellner et al., 2021; MacKenzie & Bailey, 2004). Additionally, we tested the spatial autocorrelation among model residuals for each species by survey year using Moran's *I* where values range from −1 to 1, indicating perfect dispersion to perfect clustering, with values near 0 suggesting no autocorrelation (Moran, 1948). As this is a post hoc diagnostic test based on frequentist frameworks, we considered the effect of spatial autocorrelation among model residuals to be significant if *p*‐value <0.05.

### Estimating power and simulating trends in occupancy

We conducted a spatially explicit simulation‐based power analysis, using the framework described in Southwell et al. (2019). The aim was to evaluate the power of the Glenelg Ark monitoring program to detect changes in threatened native mammals (SBB and LNP) and introduced predators (fox and feral cat) for the next 10 years under the existing design, as well as scenarios of increasing or decreasing survey effort.

The framework simulates an increase or decrease in occupancy trends over time (10 years) in baited and unbaited areas of the landscape, with the magnitude of change in occupancy defined using effect sizes. If modeling a decline, the effect size is proportional to the *initial occupancy* value of a cell, not the absolute change in occupancy. However, if modeling an increase, the effect size is proportional to the *potential increase* in occupancy (i.e., the difference between current occupancy and one), not the initial occupancy (Southwell et al., 2019). For example, if the occupancy value for a cell in the occupancy raster for a species is 0.6 at the start, a 50% decline would result in 0.30 occupancy (0.6 – [0.5 × 0.6] = 0.3) at the end of the monitoring duration (10 years), while a 50% increase would result in 0.80 occupancy (0.6 + 0.5[1–0.6] = 0.8) at the end of the monitoring duration.

**FIGURE 2:**
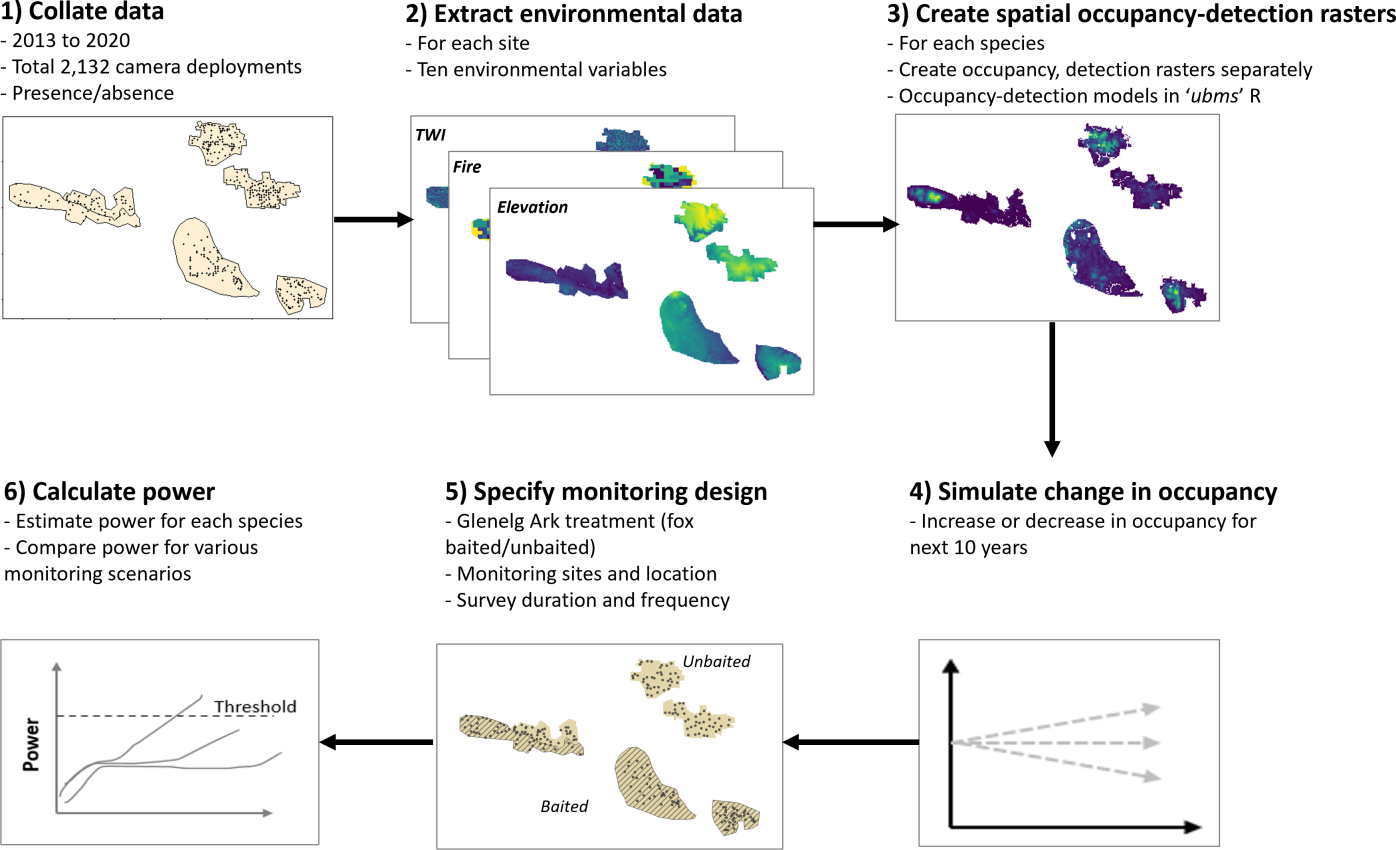
Conceptual diagram for spatially explicit simulation framework to estimate the statistical power of Glenelg Ark monitoring program.

The simulation framework requires information on the number of monitoring sites (*n* = 240 sites for Glenelg Ark monitoring program) and their location, the intervals when monitoring occurs (i.e., yearly monitoring), the number of repeat visits to a site in a survey period (= 5 for the number of weeks surveyed), and the detection method (camera monitoring) used to survey each species. Given occupancy and detectability are spatially explicit, sites in the landscape can be specified to be at: (1) fixed locations, that is, the existing 240 monitoring sites, (2) sites removed or added at random, in addition to the fixed locations, or (3) sites added in areas of highest predicted occupancy in addition to the fixed locations. A summary of the steps required for power analysis is outlined in Figure 2.

For each scenario (increasing or decreasing effect) and monitoring design (number of sites), we simulated detection histories for our study species, as per Southwell et al. (2019). The simulated detection histories were then fitted to an occupancy‐detection model that included an effect of baiting on detectability and an interaction between year and baiting on occupancy. We also included site as a random effect on occupancy. We simulated detection histories 1000 times and calculated the statistical power as the proportion of times the coefficient for an interaction between year and baiting had credible intervals that did not overlap 0 (alpha <0.05, two‐tailed test). A monitoring design is typically considered to have sufficient power to detect change if the statistical power is >0.8 (Smart et al., 2022; Southwell et al., 2019).

### Monitoring scenarios

We estimated the power of the monitoring program to detect changes for four plausible scenarios (Table 1). The original plan of Glenelg Ark monitoring did not include aims to detect declines for threatened native mammals in unbaited areas or increases in fox occupancy in unbaited areas. However, given the low naive occupancy of threatened native mammals (9% mean occupancy for SBBs and 4% for LNPs between 2013 and 2019) and high naive fox occupancy (70% mean occupancy for foxes between 2013 and 2019) in unbaited areas, we determined it was important to evaluate the statistical power in unbaited areas as it could inform potential relocation of monitoring resources. Similarly, the aim of the monitoring program also did not include aims to detect a potential mesopredator release in feral cats in fox‐baited areas. Hence, we determined it was important to evaluate the statistical power of potential mesopredator effects—a decrease in feral cats in unbaited areas and an increase in feral cats in fox‐baited areas.

**TABLE 1:** Power of Glenelg Ark monitoring program to detect a 10%, 20%, 30%, 40% and 50% change in occupancy for four scenarios.

Scenario	Species	Occupancy in baited areas	Occupancy in unbaited areas
1	Threatened native mammals, feral cats	Increase	No change
2	Threatened native mammals, feral cats	No change	Decrease
3	Introduced predators	No change	Increase
4	Introduced predators	Decrease	No change

*Note*: For each scenario, four survey designs are tested: (1) existing monitoring design (240 sites), (2) removing 40 sites at random (200 sites), (3) adding 40 sites at random (280 sites), and (4) adding 40 sites in areas of highest predicted occupancy (maxocc 280).

For each scenario, we estimated an increase or decrease in occupancy by 10%, 20%, 30%, 40%, and 50% over a period of 10 years, and tested the statistical power of four survey designs: (1) the existing Glenelg Ark monitoring sites (240 sites), (2) if 40 sites were removed at random from the landscape (200 sites), (3) if 40 sites were added at random to the landscape (280 sites), (4) if 40 sites were added to areas of maximum predicted occupancy (280 sites). We set 40 sites as the maximum number of additional sites that could be surveyed, considering the costs and feasibility for land managers in the region (Le Duc, pers. comm).

## RESULTS

### Occupancy and detectability

There were 329 SBB (15.4% naive occupancy), 206 LNP (9.7% naive occupancy), 930 fox (43.6% naive occupancy), and 411 feral cat (19.3% naive occupancy) detections in the 2132 camera‐trap deployments over the study period, where detections are defined on a weekly basis. There was weak to no spatial autocorrelation for all study species for all survey years (highest significant Moran's *I* <0.15; Appendix S1: Table S2). Predicted occupancy for SBB, LNP, and fox by year and treatment showed similar trends to naive occupancy between 2013–2019; however, estimates for feral cats were uncertain due to very large CIs (Appendix S1: Figures S3 and S4).

**FIGURE 3:**
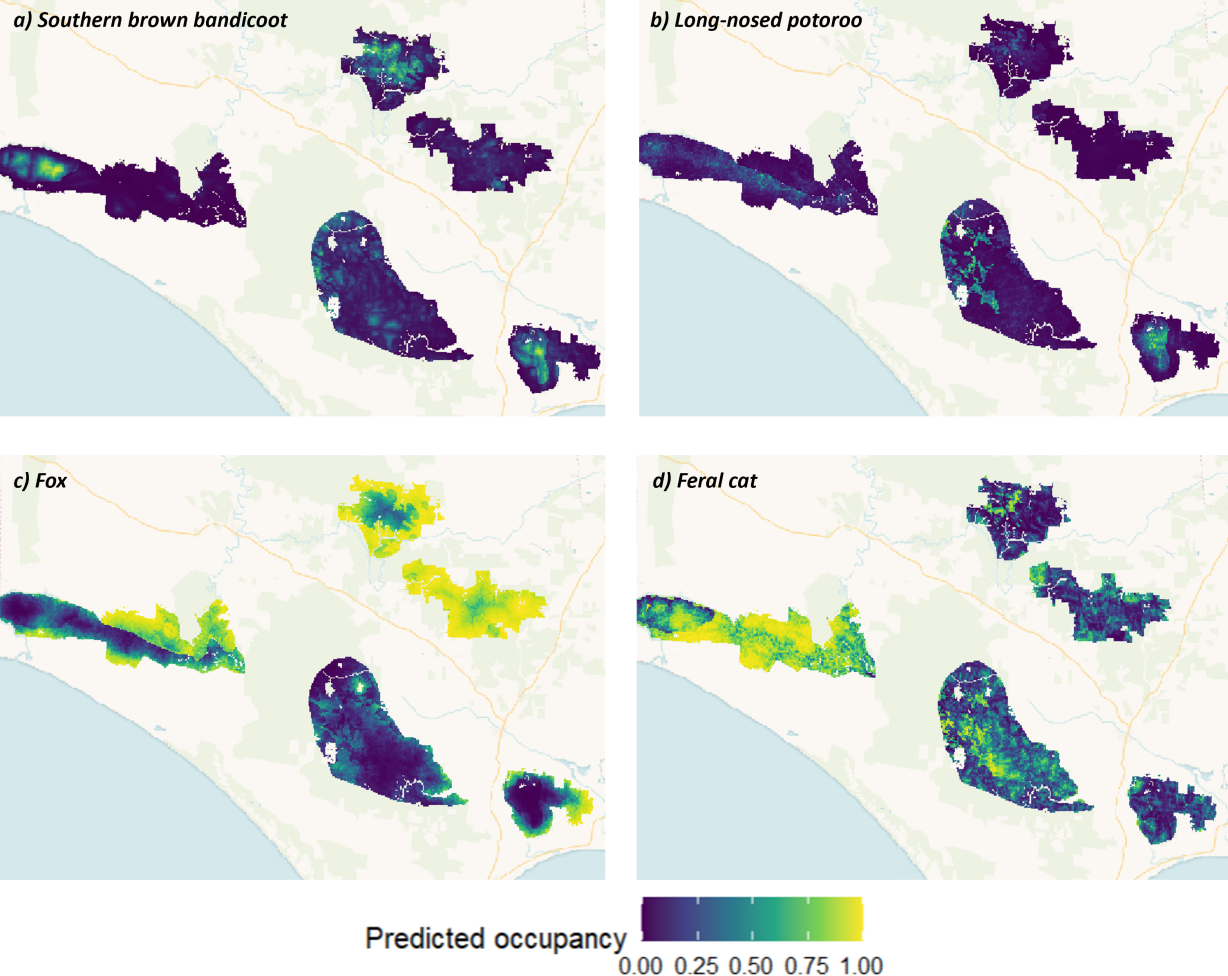
Mean predicted occupancy for (a) southern brown bandicoot, (b) long‐nosed potoroo, (c) fox, and (d) feral cat in Glenelg Ark monitoring areas (refer to Figure 1 for study area). Occupancy‐detection models included data collected from 2013 to 2020. Mean predicted detectability for study species are provided in Appendix S1: Figure S5. Predicted occupancy and detectability values range from 0 (low) to 1 (high).

**FIGURE 4:**
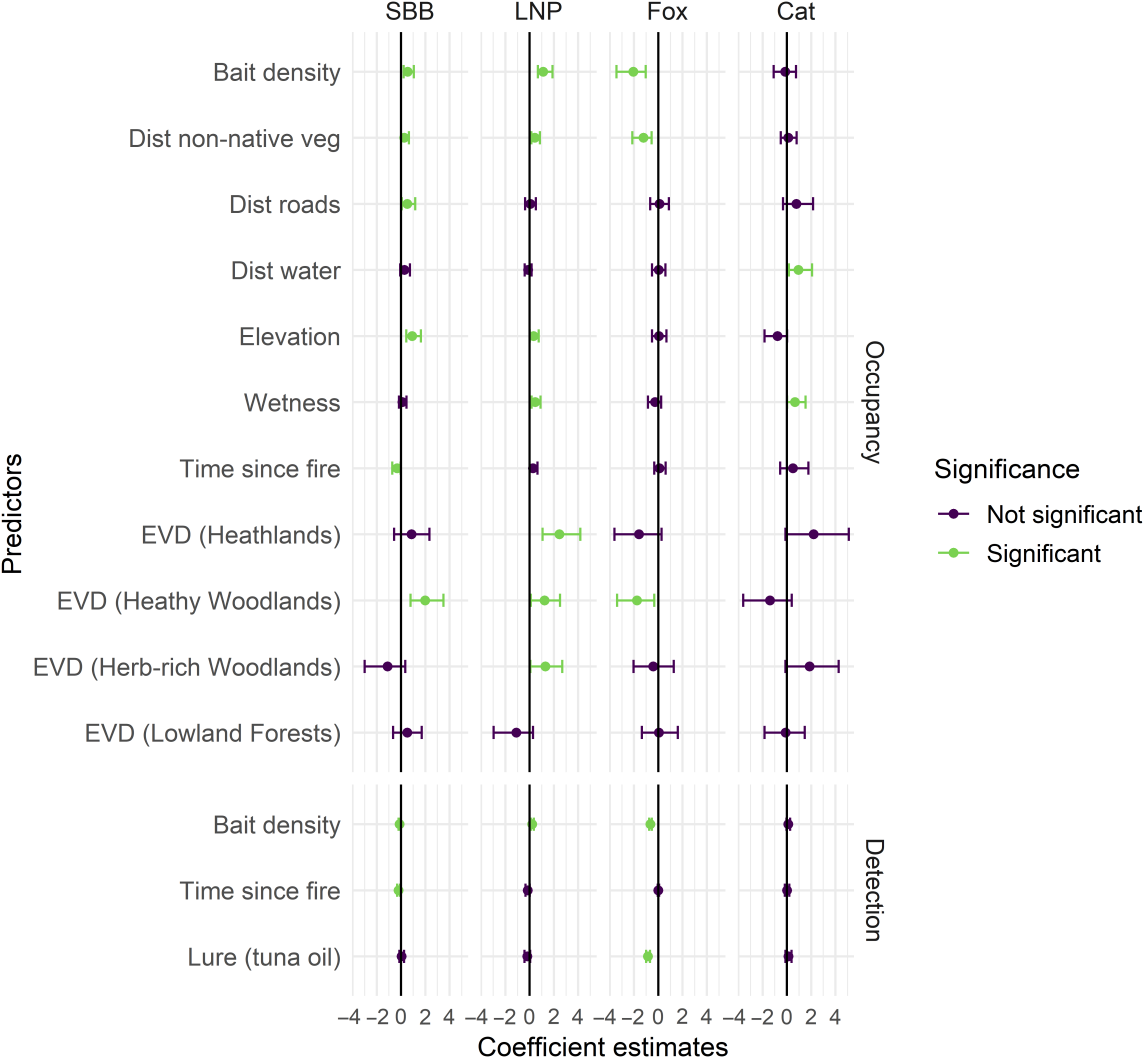
Model coefficients and 95% credible intervals for the occupancy‐detection model for each species. We ran a “full” model with bait density, time since fire, and lure type affecting detectability, and all predictors except lure type affecting occupancy. “Dry forests” was the reference level for vegetation type (EVD), and “peanut butter” for lure type. We considered that there was support for an effect of predictors on occupancy and detection when the 95% credible interval did not overlap 0. Coefficient estimates show size and direction of the effect of predictors on occupancy and detection.

Predicted occupancy for SBB ranged from 0 to 0.84 (Figure 3a). There was support for a positive effect of bait density, distance to non‐native vegetation, distance to roads, and elevation on occupancy of SBB, and a negative effect of TSF (Figure 4). Compared to dry forests, SBB occupancy was higher in heathy woodlands (Figure 4). Bait density and TSF were negatively associated with detectability of SBBs, with weekly detectability ranging from 0.22 to 0.44 (Figure 4; Appendix S1: Figure S5). Model fit for SBB was considered good (posterior predictive *p*‐value = 0.44).

Predicted LNP occupancy ranged from 0 to 0.90; however, the model fit was weak (posterior predictive *p*‐value = 0.18; Figure 3b). There was support for a positive effect of bait density, distance to non‐native vegetation, elevation, and wetness on LNP occupancy (Figure 4). Compared to dry forests, LNP occupancy was higher in heathlands, heathy woodlands, and herb‐rich woodlands (Figure 4). Similarly, bait density was positively associated with LNP detectability. Weekly detectability of LNPs was higher than that of SBBs (0.36–0.68; Appendix S1: Figure S5).

Predicted fox occupancy ranged from 0 to 0.98 with higher estimates observed in unbaited areas (Figure 3c). There was support for a negative effect of bait density on fox occupancy, along with distance to non‐native vegetation. Similarly, there was support for a lower fox occupancy in heathy woodlands compared to dry forests (Figure 4). Weekly fox detectability decreased with increasing bait density and was lower in sites with tuna oil as lure type (Figure 4). Model for foxes was considered a good fit (posterior predictive *p*‐value = 0.52).

Feral cat occupancy ranged from 0 to 0.99 (Figure 3d), however, the model fit was weak (posterior predictive *p*‐value = 0.03). Distance to water and wetness were positively associated with feral cat occupancy (Figure 4). There was some evidence that feral cat occupancy was higher in heathlands and herb‐rich woodlands than dry forests; however, the 95% credible intervals marginally overlapped zero indicating uncertainty (Figure 4). Weekly feral cat detectability was low across baited and unbaited areas (0.08–0.12; Appendix S1: Figure S5).

### Power analysis

**FIGURE 5:**
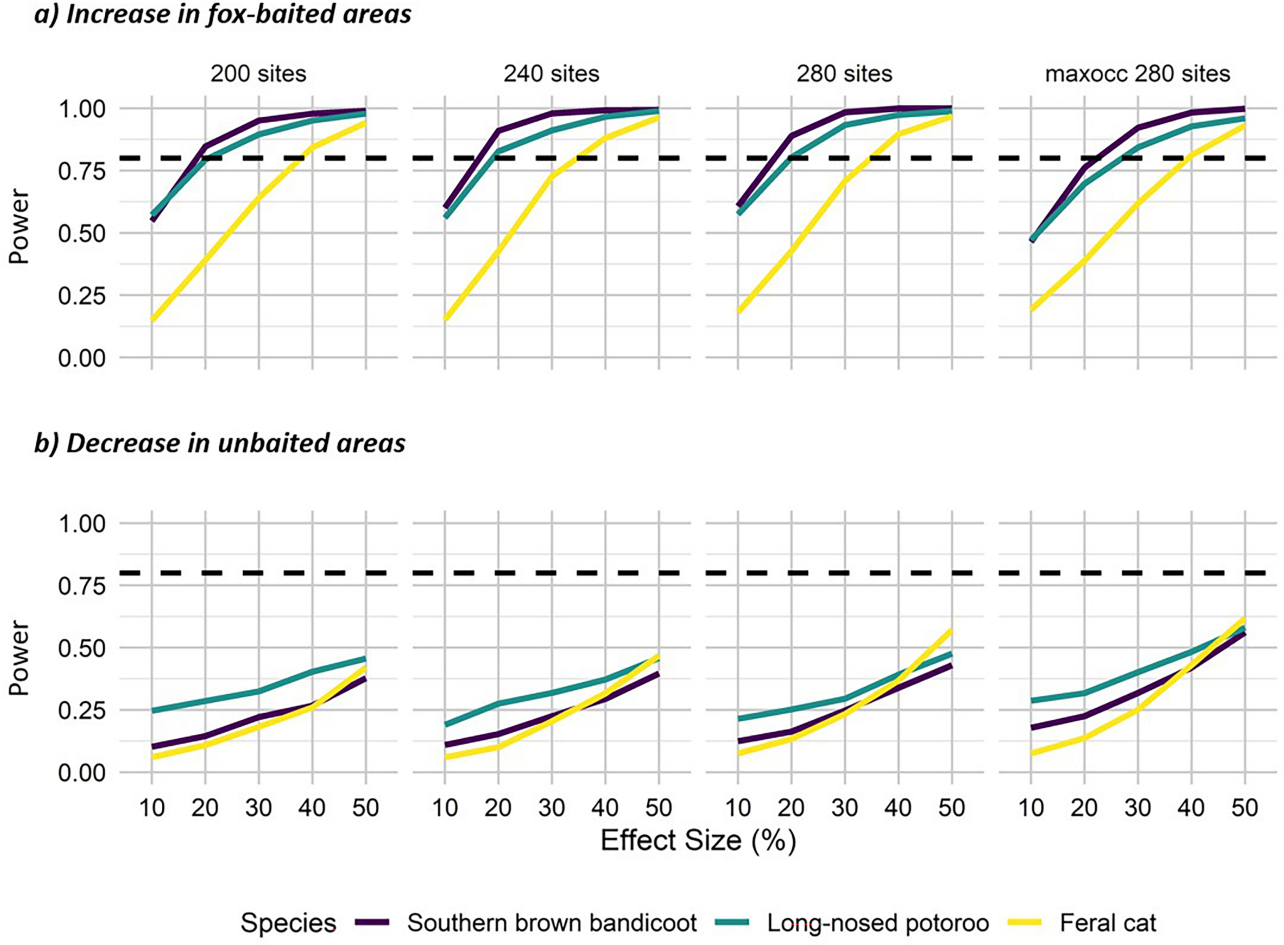
Power to detect an (a) increase in occupancy for threatened native mammals and feral cats in fox‐baited areas (assuming no change in occupancy in unbaited areas), (b) decrease in occupancy for threatened native mammals and feral cats in unbaited areas (assuming no change in occupancy in fox‐baited areas) over a 10‐year monitoring period across Glenelg Ark. Four scenarios are plotted: (1) existing monitoring design (240 sites), (2) removing 40 sites at random (200 sites), (3) adding 40 sites at random (280 sites), and (4) adding 40 sites in areas of highest predicted occupancy (maxocc 280). Horizontal dotted line represents 80% power.

Our results indicated that the current monitoring program (240 sites) had adequate power (>0.8) to detect at least a 20% increase in occupancy for each threatened native mammal (SBB and LNP) in fox‐baited areas over the next 10 years, assuming no change in effect in unbaited areas (Figure 5a). As expected, the power of the current monitoring program (240 sites) increased as effect sizes increased (Figure 5a). Removing or adding 40 sites at random (resulting in 200 or 280 sites) did not substantially vary the power to detect an increase in occupancy for SBB and LNP. Adding 40 sites in areas with the highest predicted occupancy to the existing monitoring design reduced the power to detect smaller increases in occupancy (maxocc 280; Figure 5a) because the magnitude of any increase at these sites was much less than at sites with low initial occupancy.

In contrast, the current monitoring program did not show adequate power (<0.6 for all scenarios) to detect even large decreases (50%) in occupancy of SBB and LNP in unbaited areas over a 10‐year period, assuming no change in occupancy in fox‐baited areas (Figure 5b).

The current monitoring program showed adequate power to detect a 40% or greater increase in feral cat occupancy in fox‐baited areas (Figure 5a). However, the program's power to detect decreases in feral cat occupancy in unbaited areas was low (<0.7) regardless of the survey scenario (Figure 5b).

**FIGURE 6:**
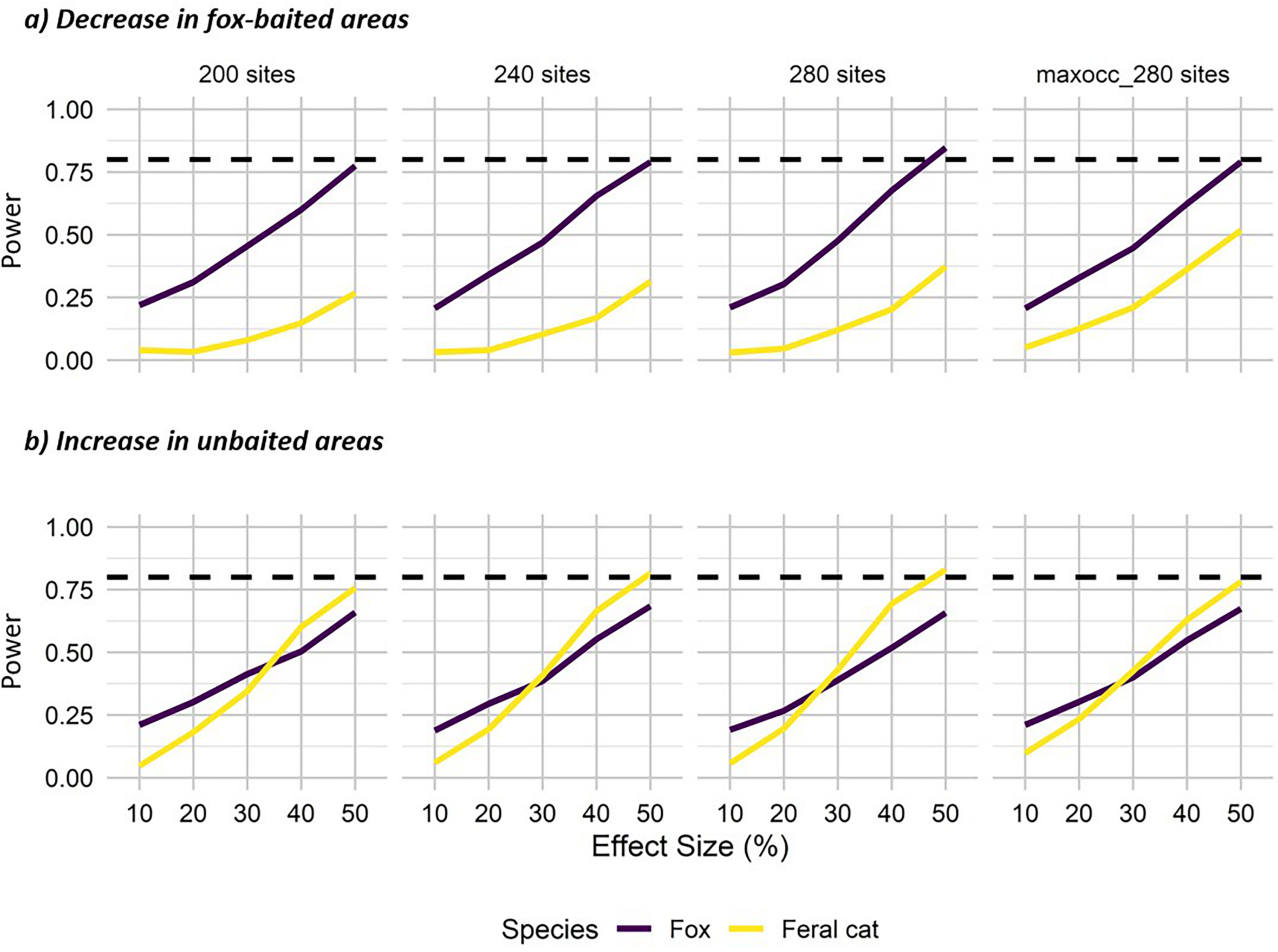
Power to detect a decrease in occupancy for (a) introduced predators in fox‐baited areas (assuming no change in occupancy in unbaited areas) and (b) increase in occupancy for introduced predators in unbaited areas (assuming no change in occupancy in fox‐baited areas) over a 10‐year monitoring period across Glenelg Ark. Four scenarios are plotted: (1) existing monitoring design (240 sites), (2) removing 40 sites at random (200 sites), (3) adding 40 sites at random (280 sites), and (4) adding 40 sites in areas of highest predicted occupancy (maxocc 280). Horizontal dotted line represents 80% power.

For foxes and feral cats, the current monitoring program (240 sites) did not show adequate power to detect even a 50% decline in occupancy in fox‐baited areas over a 10‐year period, assuming no change in occupancy in unbaited areas (power was nearing 0.8 for foxes; Figure 6a). Power declined further as effect sizes decreased (Figure 6a). Power to detect declines in occupancy in baited areas for introduced predators did not considerably vary when 40 sites were removed or when 40 sites were added to locations where predicted occupancy was high (Figure 6a). However, there was adequate power to detect a 50% decline in foxes when 40 sites were added at random to the existing monitoring design (>0.8; Figure 6a).

In contrast, the current monitoring program only had adequate power (>0.8) to detect a 50% increase in feral cats in unbaited areas over the next 10 years (Figure 6b). However, the monitoring design did not have adequate power to detect an increase in fox occupancy for the next 10 years for any of the tested effect sizes (Figure 6b). Removing or adding sites (randomly and in areas of highest predicted occupancy) did not considerably vary power for detecting fox or feral cat occupancy in unbaited areas (Figure 6b).

## DISCUSSION

Detecting temporal patterns in populations of conservation interest is fundamental to most long‐term conservation monitoring programs (Marsh & Trenham, 2008). We built species occupancy models to evaluate the effectiveness of a long‐term predator management program in southeast Australia and assess the statistical power of the associated monitoring design. Both threatened native mammal species were more likely to occupy areas with high densities of fox baits, whereas foxes were less likely to occupy these areas. We found that in fox‐baited areas, the existing monitoring program had adequate power (>0.8) to detect increases in threatened native mammal occupancy over 20% and feral cat occupancy over 40%, and declines in fox occupancy over 50%. However, in areas with no fox baiting, the program did not have adequate power to detect decreases in threatened native mammal occupancy, nor increases in feral cat or fox occupancy over the next 10 years. Our study highlights that monitoring could be potentially reallocated to improve statistical power to detect declines in unbaited areas for threatened native mammals with low occupancy, as resources for long‐term monitoring programs are often limited. Our study also highlights that additional monitoring for introduced predators is likely needed to detect a potential mesopredator release of feral cats.

### Predictors influencing threatened native mammal and introduced predator occupancy

Long‐term fox control is widely used in Australia to reduce fox predation impacts on native species and in turn improve biodiversity conservation (Saunders et al., 2010). We found that foxes were less likely to occupy areas with high densities of fox baits, whereas SBBs and LNPs had higher occupancy in these areas. This suggests that fox baiting is having the desired conservation outcomes; however, the benefits appear to be largely localized to the core of baited areas, where bait density is highest, sites are further from areas of non‐native vegetation and elevations are typically higher. Most studies on predator control through baiting typically compare baited to unbaited treatments (see review Hunter et al., 2018). However, it is often unclear whether the benefits of baiting are spatially consistent within the treatment areas. Some studies have shown little to no effect of long‐term fox baiting on native fauna (e.g., Dexter et al., 2013; Walsh et al., 2012) and further research would be useful to see whether predator and native faunal responses to baiting are localized within treated landscapes.

Although the probability of SBB and LNP occupancy was predicted to be higher in areas with high bait densities, predicted occupancy rates remained low (<7%) overall across baited and unbaited areas. Further, no consistent improvements in naive or predicted SBB and LNP occupancy in either treatment occurred between 2013 and 2019 (Appendix S1: Figures S3 and S4). It is possible that the system has reached a stable state under the current baiting program, along with external factors like habitat suitability limiting an increase in threatened native mammal occupancy in baited areas. Similarly, a potential “mesopredator release” of feral cats in fox‐baited areas could result in a net negative impact on threatened native mammals and reduce the efficacy of fox control (Doherty & Ritchie, 2017; Takimoto & Nishijima, 2022). Although there was no evidence of a relationship between bait density and feral cat occupancy in our study, this may simply reflect the weak model fit for feral cats, which is likely due to their low weekly detectability. Rees et al. (2023) modeled feral cat density instead of occupancy across the same study region and found strong support for a negative association between feral cat density and fox occupancy at a fine spatial scale (4 ha). Feral cat occupancy was high in herb‐rich woodlands within baited areas, where LNP occupancy was also predicted to be high. A combination of fox baiting and ideal habitat conditions may benefit threatened native mammals, but it could be problematic if these conditions also create more opportunities for feral cats.

The occupancy of SBBs and LNPs across our study region was not only influenced by baiting, but several other environmental variables. In contrast to foxes, SBBs and LNPs were more likely to occur further from non‐native vegetation (i.e., at sites further from cleared farmland). This may reflect a gradient in predation pressure—telemetry studies also have shown that foxes select strongly for the edges between forest and farmland in our study region (Hradsky, Robley, et al., 2017). SBBs and LNPs were also more likely to occur in areas with higher elevation, and LNPs were more likely to occur wetter sites. SBBs and LNPs display a wide range of macrohabitat preferences; several other studies have shown selection for structurally complex vegetation, potentially because it provides better protection from fox and feral cat predation (e.g., Miritis et al., 2020; Robinson et al., 2018). There was some evidence that feral cats were more likely to occur in areas of higher wetness (Doherty et al., 2014), however, the model fit for feral cat was weak and there was considerable uncertainty around the estimates.

We expected TSF to have a strong effect on threatened native mammal occupancy. Overly frequent, large, or intense fires can have long‐term negative impacts on threatened native faunal populations, particularly through reduced survival due to direct mortality or lack of resources like food and shelter (Santos et al., 2022). However, long intervals between fire can also result in unsuitable habitats for some medium‐sized native mammals that benefit from early or mid‐successional habitats (Arthur et al., 2012; Hayward et al., 2007). SBBs in our study had a negative association with TSF, possibly due to senescing shrub cover when there are long intervals between fire (e.g., Arthur et al., 2012). In contrast, there was no evidence for an effect of TSF on occupancy for LNPs or introduced predators, potentially because these relationships are nonlinear (Rees et al., 2024). Incorporating polynomial or regression splines could help in identifying nonlinear relationships between TSF and species occurrence in future research. Impacts of introduced predators in recently burned areas have been well documented, however, knowledge on how long‐term fire patterns affect introduced predators are relatively limited (Hradsky, 2020) and may vary between vegetation types (Rees et al., 2024).

Incorporating fine‐scale habitat preferences of threatened native mammals and introduced predators across the management region will help improve prioritization of management and monitoring resources. For example, less‐monitored areas with high predicted native mammal occupancy could be targeted for surveys and help validate predictive accuracy of the models. Additionally, predator control could be intensified in areas where occupancy rates are currently low but environmental variables indicate that habitat suitability for priority threatened species would be high if bait density increased, such as heathy woodlands and locations distant from non‐native vegetation.

### Monitoring scenarios

The monitoring design had adequate power to detect increases for the target threatened native mammals, SBBs and LNPs, in fox‐baited areas. For SBBs and LNPs, the existing monitoring program was more likely to detect increasing trends in fox‐baited areas compared to decreasing trends in unbaited areas. The differences in power for increasing and decreasing trends were due to discrepancies in the way the framework calculates effect sizes. For declining trends, the effect size is considered proportional to the initial occupancy value, whereas for increasing trends, the effect size is proportional to the potential increase in occupancy. Given SBB and LNP occupancy were low in both baited and unbaited areas (Appendix S1: Figures S3 and S4), a percentage increase in occupancy resulted in larger magnitude changes than the same percentage decline, an important note to be made within the spatially explicit power analysis used here (Southwell et al., 2019).

As expected, the monitoring design was sensitive to the magnitude of increase or decrease in occupancy of interest and, to a lesser extent, the number of survey sites. However, removing or adding 40 sites at random, or adding 40 sites to areas of highest predicted occupancy had little effect on the monitoring program's power to detect changes in SBB or LNP occupancy. These additional resources could be reallocated as removing sites does not substantially alter the study's statistical power to detect changes in threatened native mammals. For example, new areas could be monitored to increase representation of environmental predictors that are considered significant for SBB and LNP occupancy (Robley et al., 2023).

The existing Glenelg Ark monitoring design did not have adequate power (<0.8) to detect even large declines in baited areas for feral cats, and only near‐adequate statistical power in detecting a 50% decline in occupancy for foxes for most scenarios. Fox and feral cat occupancy have not considerably varied in the last 10 years (Appendix S1: Figures S3 and S4), suggesting that significant changes are unlikely under the current management regime. Nonetheless, the ability to detect changes is important, particularly if new management actions are included, for example, introducing baiting in areas with high habitat suitability for threatened native mammals or introducing feral cat management.

Glenelg Ark was primarily set up to control foxes to improve native mammal occupancy. However, recent studies have shown a positive association in feral cat occupancy in fox‐controlled areas (e.g., Marlow et al., 2015; Rees et al., 2023), which is likely to reduce the net efficacy of fox control for native mammals that are vulnerable to both predator species. The monitoring design had adequate power to detect large increases in feral cat occupancy in fox‐baited areas (>40%); however, power to detect declines for feral cats in baited areas was lower compared to foxes, due to their low median weekly detectability (feral cat = 0.1, fox = 0.18) and possibly the lack of monitoring sites in areas of higher predicted feral cat occupancy. Our study underscores the challenges in detecting potential mesopredator release of feral cats; even with 240 monitoring sites, detecting a doubling of feral cat occupancy was unlikely. Consequently, more intensive monitoring is necessary to make accurate assessments around mesopredator release effects.

### Recommendations for improving monitoring efforts

Increasing the intervals between surveys and reallocating the resources to survey additional sites could increase power to detect declines for threatened species with low occupancy in unbaited areas. Given the low naive and predicted occupancy of SBB and LNP (Appendix S1: Figures S3 and S4), it could be beneficial to survey a different set of sites biannually to cover more areas in the landscape. A review of a long‐term faunal monitoring program in northern Australia by Einoder et al. (2018) indicated that low statistical power to detect declines in occupancy could be improved by changing the allocation of effort (i.e., increasing the number of monitoring sites and reducing the frequency of monitoring). If removing sites from an existing monitoring design does not considerably vary power, these resources could perhaps instead be used to (1) manage and monitor another pair of baited/unbaited areas in the landscape or (2) survey areas with poorly understand combinations of environmental variables (e.g., areas where predicted occupancy is high due to a particular vegetation type or higher elevations). Further research to create a cost model which links the number of monitoring sites and survey frequency to a total budget could help optimize the survey design for a given monitoring budget (Smart et al., 2022).

## CONCLUSION

Designing large‐scale, long‐term monitoring programs often involves a trade‐off between maximizing the probability of detecting a species at a site and increasing the number of monitoring sites (Einoder et al., 2018; Field et al., 2005). It is important that monitoring is carried out in a way that enables inference about the entire area of interest based on the sampled sites (Pollock et al., 2002). The current monitoring design could be improved by further random stratification of monitoring sites to incorporate environmental variables like vegetation type or higher elevations that may provide more suitable habitats for threatened native mammals. Additional monitoring and resource allocation will depend on whether land managers’ primary goal is to detect changes in threatened species that are restricted to highly localized habitats, or whether the goal of the monitoring is to monitor changes in widespread species on a landscape scale (Southwell et al., 2019). As our study shows, assessing the statistical power for existing monitoring programs using spatially explicit power analysis is important for ensuring monitoring strategies are robust and cost‐effective. We show that the current monitoring design is adequate to detect increases in threatened native mammals in fox‐baited areas, but lacks power to detect decreases in foxes or any changes in feral cat occupancy.

Our results show that the baiting program is effective in “core areas” where bait density is high, that is, fox occupancy is lower and threatened native mammal occupancy is higher in areas of high bait density. However, the benefits of the baiting program appear spatially patchy, and it is plausible that the system has reached a stable state under the current management program. Given the statistical power of the existing design is adequate to detect small increases in threatened native mammal occupancy in baited areas, the lack of a strong signal of increasing occupancy is likely to reflect the true state, rather than a lack of statistical power. Consequently, priority should be given to increasing management efficacy, rather than increasing monitoring effort, if land managers want to achieve further biodiversity gains. For example, bait density could be increased in areas where our modeling shows high habitat suitability for threatened native mammals. However, additional monitoring of predator populations is likely to be needed to adequately assess the effectiveness of predator management programs and to detect any potential mesopredator release of feral cats.

## AUTHOR CONTRIBUTIONS

Study conceptualization and design: Vishnu Menon, Bronwyn A. Hradsky. Funding acquisition: Bronwyn A. Hradsky, Brendan Wintle. Collection of previously published data: Vishnu Menon. Additional datasets contributed by Alan Robley and Matthew W. Rees. Data curation: Vishnu Menon. Formal analysis: Vishnu Menon, with input from Darren Southwell and David P. Wilkinson. Writing – original draft preparation: Vishnu Menon. Writing – review and editing: Vishnu Menon, Bronwyn A. Hradsky, Alan Robley, Matthew W. Rees, Darren Southwell, David P. Wilkinson, Katherine Giljohann, and Jack Pascoe.

## CONFLICT OF INTEREST STATEMENT

The authors declare no conflicts of interest.

## Supporting information


Appendix S1.

## Data Availability

Data and code (Menon et al., 2026) are available in Dryad at https://doi.org/10.5061/dryad.80gb5mm4h.
