# Supplementary material for: Spatially explicit power analysis reveals challenges for a long‐term threatened species monitoring program in Australia
Source: Ecol Appl. 2026 Jun 5;36(4):e70271. doi: 10.1002/eap.70271 (PMC13238438; doi:10.1002/eap.70271)
Supplement: Supplementary file 1 — Appendix S1. [file EAP-36-e70271-s001.pdf]

<u>Appendix S1</u>

Spatially explicit power analysis reveals challenges for a long-term threatened species monitoring program in Australia

Vishnu Menon, Darren Southwell, Alan Robley, Matthew W. Rees, David P. Wilkinson, Katherine Giljohann, Jack Pascoe, Brendan Wintle, Bronwyn A. Hradsky

Ecological Applications

Section S1: Environmental predictors used in the occupancy-detection models

Table S1: Summary of the environmental predictors incorporated in the occupancy-detection models for threatened native mammals and introduced predators.

Predictor	Resolution	Range (unit)	Source	Justification
Distance to non-native vegetation	25m	0 - 4521 (m)	Inverting extent of native vegetation (Department of Environment, Land, Water & Planning 2019)	Foxes and feral cats are known to prefer edges between forests and cleared land (e.g., Hradsky et al. 2017b; McGregor et al. 2015)
Distance to roads	50m	0 - 1637 (m)	Department of Environment, Energy and Climate Action 2023	Foxes and feral cat activity have been recorded to be higher near roads (Raiter et al. 2018)
Distance to water	50m	0 - 3464 (m)	Department of Environment, Energy and Climate Action 2023	Watercourses may influence bandicoot occupancy (Catling, Burt, and Forrester 2002) and long-nosed potoroo occupancy (Robley, Moloney, and Le Duc 2023)
Normalised difference vegetation index (NDVI)	5km	0.38 - 0.51	Calculated mean annual NDVI from monthly mean NDVI layers between 2001-2020 (Bureau of Meteorology 2021)	Productivity of natural areas are hypothesised to be positively associated to native mammal abundance (White et al. 2022; Youngentob et al. 2015)
Elevation	10m	0.3 - 184.1 (m)	(Department of Environment, Land, Water & Planning 2020b)	Elevation can influence rainfall, moisture and temperature gradients, potentially affecting species occupancy in our study region (Rees et al. 2024)
Topographic wetness index	25m	5.4 - 15.8	Commonwealth Scientific and Industrial Research Organisation (Gallant and Austin 2012)	Key food sources for long-nosed potoroos and southern brown bandicoots, such as subterranean invertebrates and fungi, are impacted by soil moisture levels estimated through the wetness index (Lobert 1990; Nuske et al. 2017)
Bait density	50m	0 - 1.26 (baits per sq. km in a 2.3 km radius)	Average known maximum distance travelled by a fox from their home-range in our study region is 2.3 km (Hradsky et al. 2017b). We calculated bait density per sq. km within a 2.3 km radius of each raster cell, as per (Rees et al. 2024). Bait station locations sourced from Department of	Landscape-scale fox control in our study region is known to reduce fox occupancy (Robley et al. 2014), increase feral cat density due to a potential mesopredator release (Rees et al. 2023) and increase native

			Environment, Energy and Climate Action 2023	mammal occupancy (Robley et al. 2014)
Time since fire (TSF)	50m	0 – 85 (years)	Department of Environment, Energy and Climate Action 2023	Fires can promote introduced predator occupancy and decrease habitat suitability for threatened native mammals due to reduced vegetation cover (Hradsky 2020; Hradsky et al. 2017a)
Ecological vegetation division (EVD)	50m	Dry forests, heathlands, heathy woodlands, herb-rich woodlands and lowland forests	(Department of Environment, Land, Water & Planning 2020a)	occupancy of introduced predator and threatened native mammals are known to vary across vegetation types in south-east Australia (Rees et al. 2024)
Lure type	50m	Tuna oil or peanut butter		We wanted to account for any effect of the types of lures on occupancy and detectability

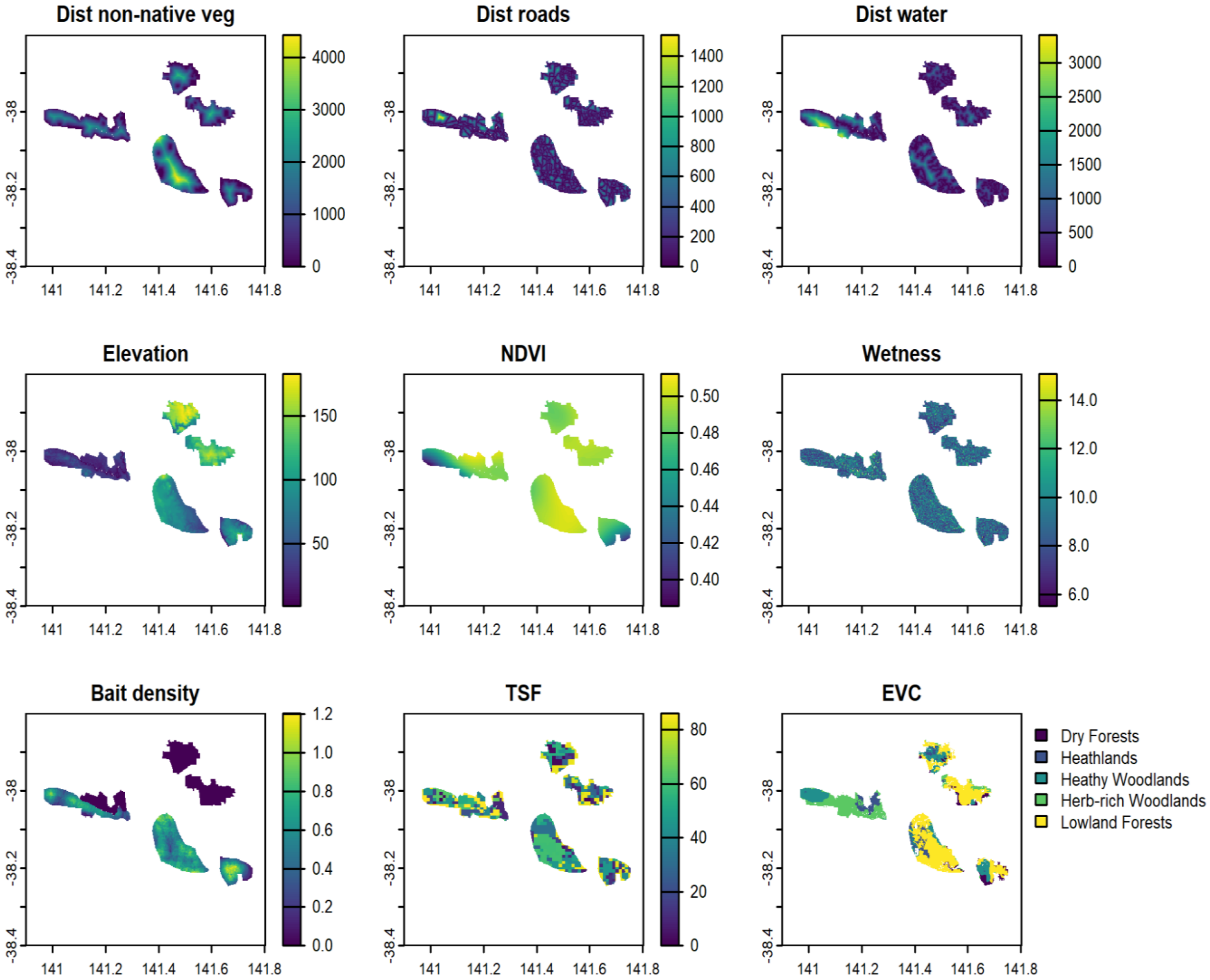

Figure S1: Environmental predictors used for building occupancy and detectability rasters.

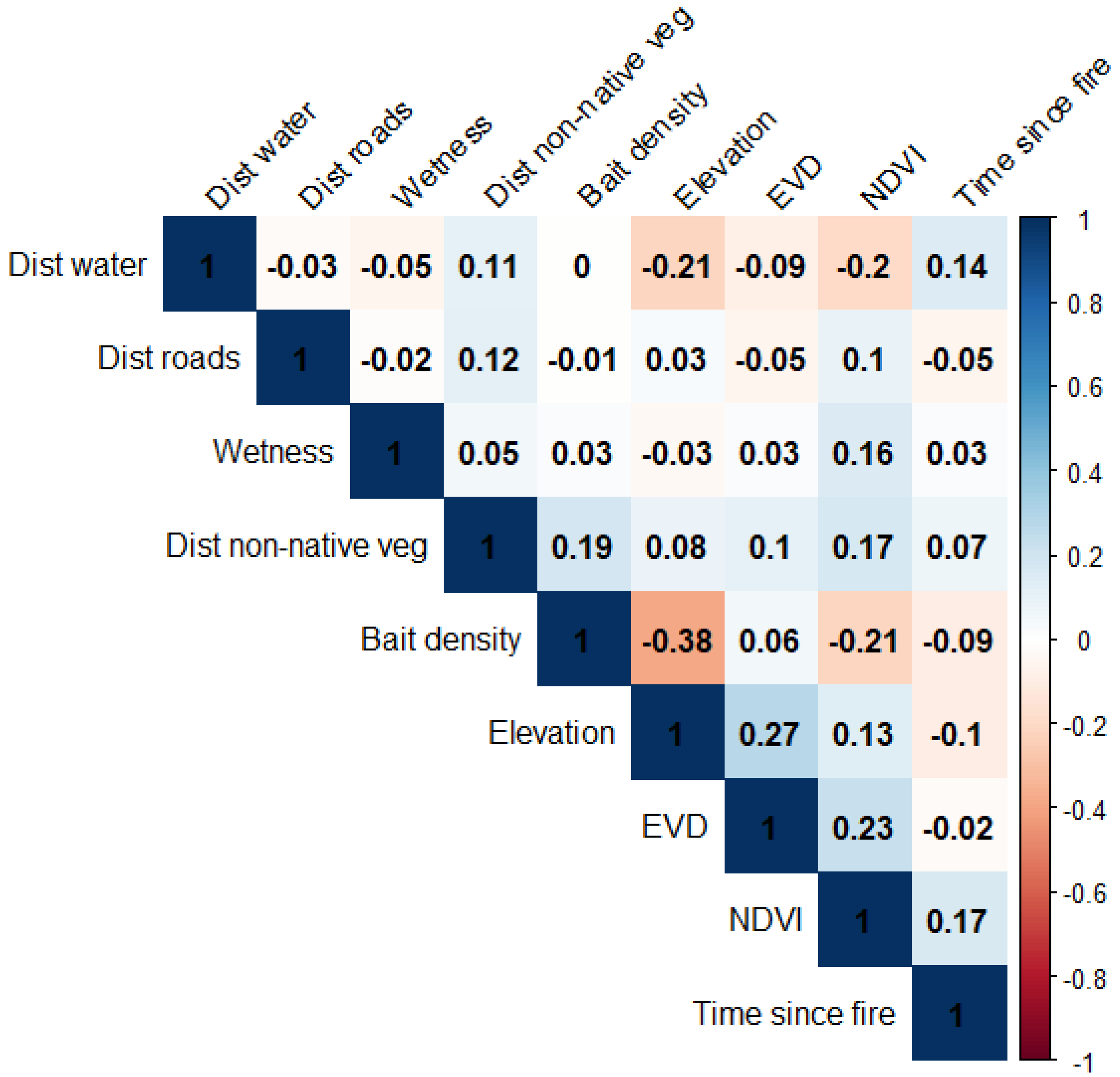

Figure S2: Correlation matrix for environmental covariates used in the occupancy-detection model.

Literature cited:

Bureau of Meteorology. 2021. *Climate Data Online.* Retrieved February 22, 2021 (www.bom.gov.au/climate/data/index.shtml).

Catling, P. C., R. J. Burt, and R. I. Forrester. 2002. “Models of the Distribution and Abundance of Ground-Dwelling Mammals in the Eucalypt Forests of North-Eastern New South Wales in Relation to Environmental Variables.” *Wildlife Research* 29(3):313. doi: 10.1071/WR01005.

Department of Environment, Energy and Climate Action. 2023. *Forest Fire Management Victoria.* Retrieved (ffm.vic.gov.au).

Department of Environment, Land, Water & Planning. 2019. “Native Vegetation - Modelled 2005 Ecological Vegetation Classes (with Bioregional Conservation Status).”

Department of Environment, Land, Water & Planning. 2020a. “Bioregions and EVC Benchmarks.”

Department of Environment, Land, Water & Planning. 2020b. “Vicmap Elevation DEM 10m.”

Gallant, John, and Jenet Austin. 2012. “Topographic Wetness Index Derived from 1" SRTM DEM-H.”

Hradsky, Bronwyn A. 2020. “Conserving Australia’s Threatened Native Mammals in Predator-Invaded, Fire-Prone Landscapes.” *Wildlife Research* 47(1):1. doi: 10.1071/WR19027.

Hradsky, Bronwyn A., Craig Mildwaters, Euan G. Ritchie, Fiona Christie, and Julian Di Stefano. 2017a. “Responses of Invasive Predators and Native Prey to a Prescribed Forest Fire.” *Journal of Mammalogy* 98(3):835–47. doi: 10.1093/jmammal/gyx010.

Hradsky, Bronwyn A., Alan Robley, Ray Alexander, Euan G. Ritchie, Alan York, and Julian Di Stefano. 2017b. “Human-Modified Habitats Facilitate Forest-Dwelling Populations of an Invasive Predator, *Vulpes Vulpes*.” *Scientific Reports* 7(1):12291. doi: 10.1038/s41598-017-12464-7.

Lobert, B. 1990. “Home Range and Activity Period of the Southern Brown Bandicoot (*Isoodon Obesulus*) in a Victorian Heathland.” Bandicoots and Bilbies, pp 319–25.

McGregor, Hugh, Sarah Legge, Menna E. Jones, and Christopher N. Johnson. 2015. “Feral Cats Are Better Killers in Open Habitats, Revealed by Animal-Borne Video” edited by B. L. Allen. *PLOS ONE* 10(8):e0133915. doi: 10.1371/journal.pone.0133915.

Nuske, S. J., K. Vernes, T. W. May, A. W. Claridge, B. C. Congdon, A. Krockenberger, and S. E. Abell. 2017. “Redundancy among Mammalian Fungal Dispersers and the Importance of Declining Specialists.” *Fungal Ecology* 27:1–13. doi: 10.1016/j.funeco.2017.02.005.

Raiter, Keren G., Richard J. Hobbs, Hugh P. Possingham, Leonie E. Valentine, and Suzanne M. Prober. 2018. “Vehicle Tracks Are Predator Highways in Intact Landscapes.” *Biological Conservation* 228:281–90. doi: 10.1016/j.biocon.2018.10.011.

Rees, Matthew W., Jack H. Pascoe, Mark Le Pla, Alan Robley, Emma K. Birnbaum, Brendan A. Wintle, and Bronwyn A. Hradsky. 2023. “Mesopredator Release among Invasive Predators: Controlling Red Foxes Can Increase Feral Cat Density and Alter Their Behaviour.” *Journal of Applied Ecology* 1365-2664.14402. doi: 10.1111/1365-2664.14402.

Rees, Matthew W., Brendan A. Wintle, Alan Robley, Jack H. Pascoe, Mark Le Pla, Emma K. Birnbaum, and Bronwyn A. Hradsky. 2024. “Fox Control and Fire Influence the Occurrence of Invasive Predators and Threatened Native Prey.” *Biological Invasions* 26(3):685–703. doi: 10.1007/s10530-023-03200-6.

Robley, A., P. D. Moloney, and E. Le Duc. 2023. "Glenelg Ark - 2022 Monitoring and Evaluation Update. Arthur Rylah Institute for Environmental Research Technical Report Series No. 358". Department of Energy, Environment and Climate Action, Heidelberg, Victoria.

Robley, Alan, Andrew M. Gormley, David M. Forsyth, and Barbara Triggs. 2014. “Long-Term and Large-Scale Control of the Introduced Red Fox Increases Native Mammal Occupancy in Australian Forests.” *Biological Conservation* 180:262–69. doi: 10.1016/j.biocon.2014.10.017.

White, John G., Jacinta Sparrius, Tomas Robinson, Susannah Hale, Luke Lupone, Tom Healey, Raylene Cooke, and Anthony R. Rendall. 2022. “Can NDVI Identify Drought Refugia for Mammals and Birds in Mesic Landscapes?” *Science of The Total Environment* 851:158318. doi: 10.1016/j.scitotenv.2022.158318.

Youngentob, Kara N., Hwan-Jin Yoon, John Stein, David B. Lindenmayer, and Alex A. Held. 2015. “Where the Wild Things Are: Using Remotely Sensed Forest Productivity to Assess Arboreal Marsupial Species Richness and Abundance” edited by L. Beaumont. *Diversity and Distributions* 21(8):977–90. doi: 10.1111/ddi.12332.

Section S2: Naive and predicted species occupancy

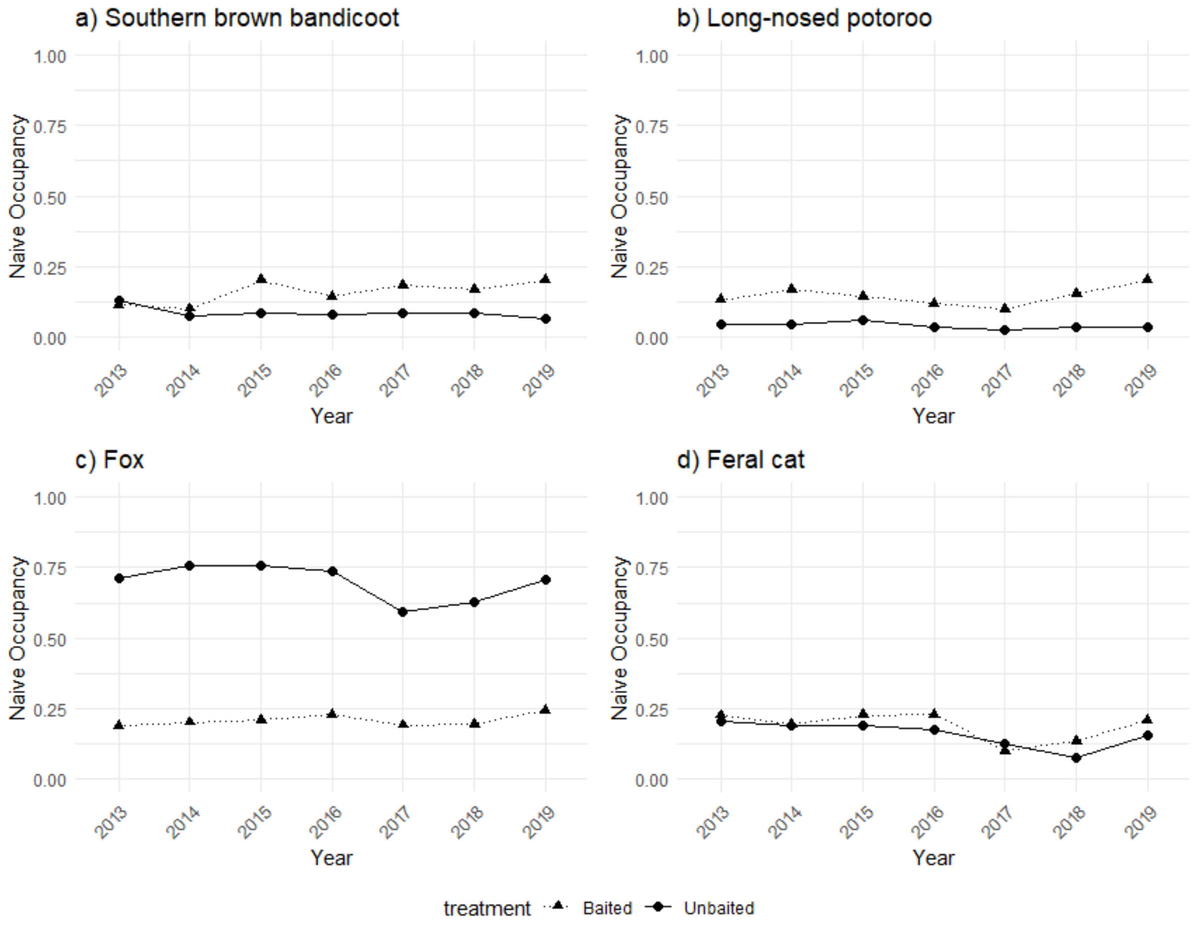

Figure S3: Naive occupancy for two threatened native mammals (southern brown bandicoot and long-nosed potoroo) and introduced predators (fox and feral cat) in fox-baited and unbaited areas in Glenelg Ark

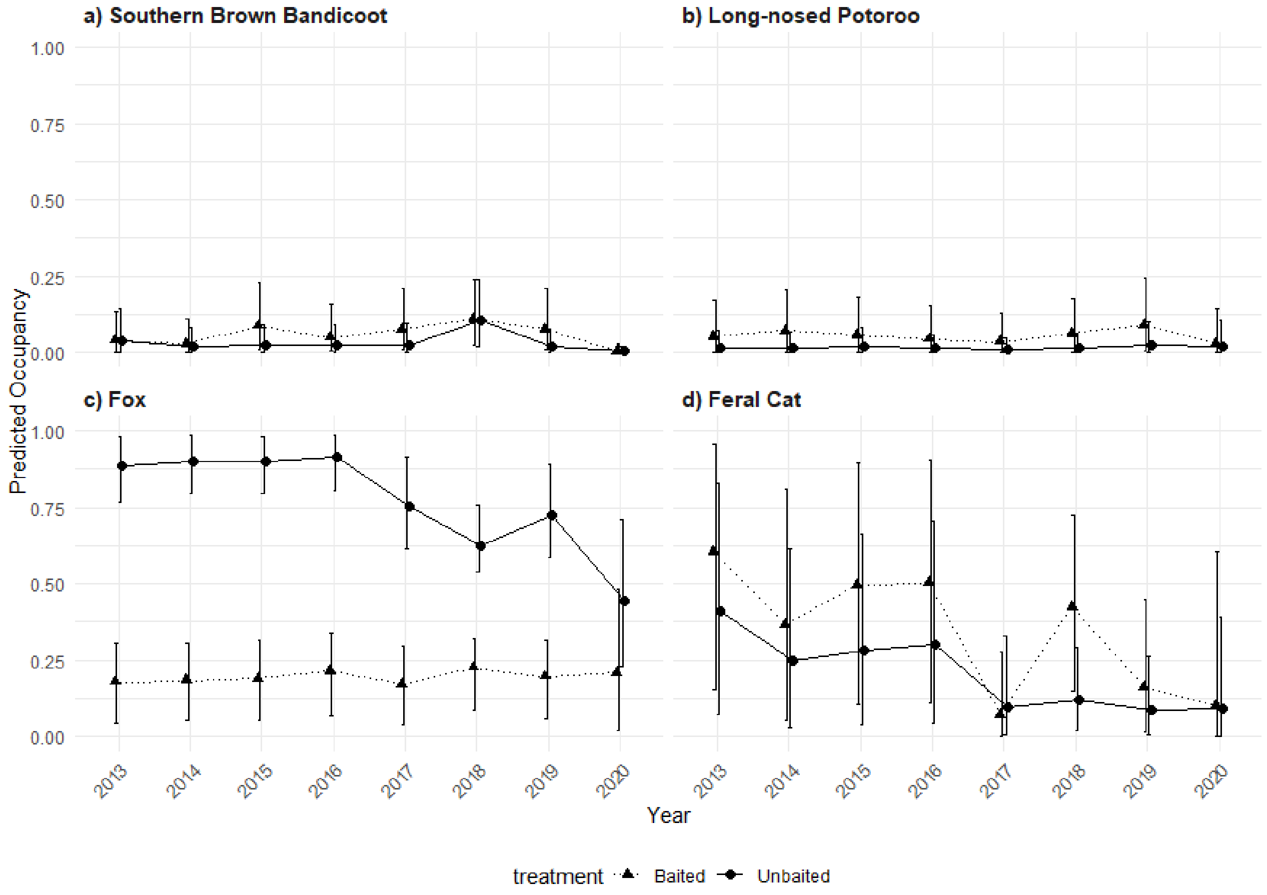

Figure S4: Predicted site occupancy estimates from occupancy-detection model with year and treatment affecting occupancy. Year 2020 only had 61 survey sites compared to a minimum of 213 sites from 2013-2019 resulting in higher uncertainty for most species (except SBB).

Section S3: Spatial autocorrelation among model residuals for the occupancy-detection models

Table S2: Moran's I value and p-values for spatial autocorrelation among model residuals by species and survey year. Moran's I values range from -1 to 1, indicating perfect dispersion to perfect clustering, with values near 0 suggesting no autocorrelation. p-value < 0.05 means a statistically significant effect. Values highlighted in bold show a statistically significant spatial autocorrelation; note that Moran's I value for these data were still low $|\leq 0.15|$.

Year	Species	Moran's I	p-value
2013	Southern brown bandicoot	0.01	0.34
2013	Long-nosed potoroo	-0.06	0.88
**2013**	**Fox**	**0.07**	**0.05**
2013	Cat	0.03	0.20
2014	Southern brown bandicoot	0.04	0.13
2014	Long-nosed potoroo	-0.05	0.85
2014	Fox	0.01	0.40
2014	Cat	-0.02	0.67
2015	Southern brown bandicoot	0.06	0.06
2015	Long-nosed potoroo	-0.03	0.70
2015	Fox	0.05	0.11
2015	Cat	0.01	0.40
2016	Southern brown bandicoot	0.05	0.11
2016	Long-nosed potoroo	-0.06	0.91
**2016**	**Fox**	**0.13**	**< 0.01**
2016	Cat	-0.02	0.64
2017	Southern brown bandicoot	0.06	0.08
2017	Long-nosed potoroo	-0.02	0.64
2017	Fox	-0.04	0.81
2017	Cat	0.04	0.17
**2018**	**Southern brown bandicoot**	**0.08**	**< 0.01**
**2018**	**Long-nosed potoroo**	**0.09**	**< 0.01**
**2018**	**Fox**	**0.06**	**0.01**
2018	Cat	0.03	0.10
**2019**	**Southern brown bandicoot**	**0.15**	**< 0.01**
2019	Long-nosed potoroo	-0.02	0.62
2019	Fox	0.04	0.13
2019	Cat	-0.01	0.56
2020	Southern brown bandicoot	0.08	0.12
2020	Long-nosed potoroo	0.05	0.21
2020	Fox	0.08	0.11

2020	Cat	-0.04	0.60

Section S4: Predicted detectability maps

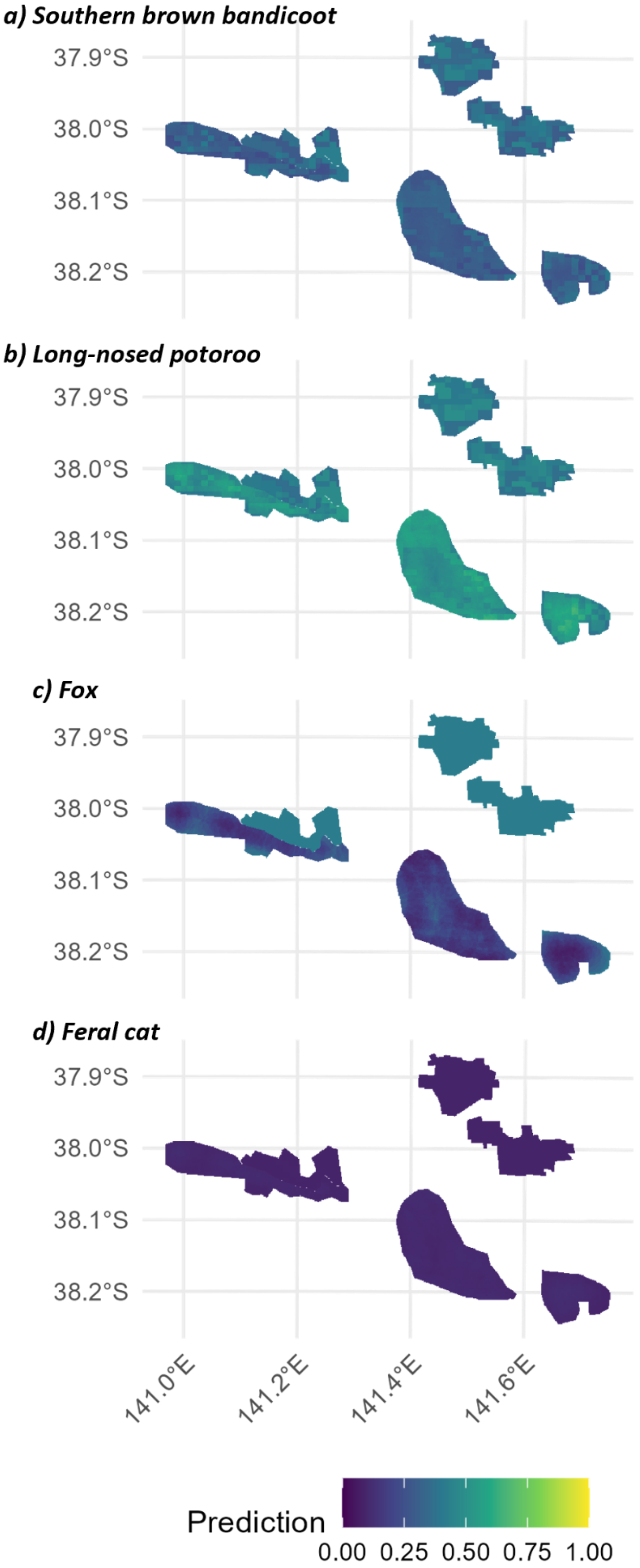

Figure S5: Predicted detectability for a) SBB, b) LNP, c) fox and d) feral cat in Glenelg Ark monitoring areas from the 'full' occupancy-detectability model.
